# Woman in Dentistry

**Published:** 1887-11

**Authors:** Jennie Kollock Hilton

**Affiliations:** Ft. Atkinson, Wis.


### THE DENTAL REGISTER.

VOL. XLI.] NOVEMBER, 1887. [No. 11.

### COMMUNICATIONS.

### Woman in Dentistry.

BY JENNIE KOLLOCK HILTON, D.D.S., FT. ATKINSON, WIS.

Prominent among the many new occupations opened to women during the past few years is the profession of dental surgery. In keeping with the enlightened spirit of the age, the question of sex in labor is being lost sight of, in the vastly more important consideration of the quality of the labor. It is the work *per se*, not the work *per sex*, that is commanding the attention of an educated and discriminating public. The question therefore naturally arises, is woman fitted for this new field of labor? Is she endowed by nature with those qualities, physical and intellectual, that will enable her to take a place in one of the most honorable and useful avocations to which the laborer can be called?

The successful operator in this field of work must bring to it, as to all other professional labor, a good degree of strength, vitality, and powers of endurance. In addition to this there is required such proficiency in mechanical skill as will enable the practitioner to operate with mathematical precision.

The intellectual qualifications demanded are of a high order. The curriculum of the dental colleges prescribes a thorough course of study, including the fundamental principles upon which the practice of dentistry is founded.

Now not all women, no more than all men, are qualified by nature, or can be qualified by virtue of a diploma, for the work of a practitioner in this field. But that some women, like some men, are thus qualified none can question who are conversant with the record of those who have attained a professional standing by virtue of the large measure of success that has attended their efforts.

With the time-worn charge—physical disqualification—urged against women entering this and other professions and arenas of employment formerly filled by men only, we will simply pause to say that for the physically disqualified (and they are legion) their entrance upon this profession is simply a physical impossibility; hence, all fears may be quieted on this score. As to the second essential qualification, namely, inventive genius, the hundreds of patents granted to women, the object of which is to lighten and facilitate labor, especially those relating to household labor, are sufficient guarantee of the fact that this creative gift has not been wholly withheld from womankind.

A prospective student of the Michigan Dental College was asked by one of the distinguished members of the profession: "Can you run a sewing machine, adjust a new needle, thread a bobbin," etc. "Oh, yes," was the rejoiner, "for that is a woman's work, you know." "Well," continued the professor, "being a woman, and understanding what is called woman's work, is a good assurance that you are not lacking in that kind of inventive genius that is essential to the practice of dental surgery."

Third, as to woman's intellectual grasp of the profession and mental qualifications as to the duties of the office, the questioning world can safely trust the verdict to the dental college which takes her training in charge, and which grants or refuses its diploma not on the question of sex but on the merits of the student.

But aside from these there are other qualifications which are as essential to the highest success of a dentist as are those already enumerated, qualities which pertain almost exclusively to woman, and which entitle her claims to a consideration by every thoughtful, fair-minded person. Prominent among these is that untiring patience that never ceases in the effort to overcome the ignorant prejudices and much needless fright of those who come under her professional care. The quick sympathy that soothes and quiets and reassures the nervous and suffering patient, the delicate and magnetic touch of woman's hand, that lessens rather than intensifies the pain consequent upon the manipulation of the relentless instruments, are essential qualities possessed by women. Qualities such as these are not the actual requirements of the curriculum, but contribute largely to that quality of success characteristic of women only. In this connection we can overlook the universally conceded intuitional power which is peculiar to the feminine mind, quickly meeting emergencies in dental practice, and rapidly arriving at a correct diagnosis of obscure and perplexing conditions.

A careful examination of the dental ledgers of some of the leading practitioners shows that a vast majority of the patients who visit the dental rooms are women and children. What more proper or more fitting than that they should pass into the hands of a skillful, sympathetic, and conscientious woman. It is now understood by dentists that the care and preservation of the deciduous or baby teeth is of equal importance with that of the second or permanent set, because their preservation not only saves the little ones much needless pain, but contributes greatly to the health of the child, and also gives us the best assurance of the regular arrangement and perfect structure of the permanent set. In this department of dental surgery there is not only room for the woman practitioner, but there is an imperative demand for the peculiar skill, tact, and sympathy that pertain to the woman dentist, by which she is enabled to successfully cope with this special class of cases.

In a letter recently received from Dr. J. Taft, of the Ann Arbor Dental College, he says: "The practice for ladies, and especially for children, is just the field for lady practitioners in dentistry. In it they can do much better than men, and it ought in the main to be done by women. There have been fifteen ladies graduated in our dental college, and I am proud of them all."

In the preparation of this paper I entered into a correspondence with the deans of the leading dental colleges throughout the United States in respect to their attitude toward women in this profession. The replies were of the most gratifying character. The institutions that had admitted them are enthusiastic in their reports, the universal verdict being they are faithful students, they make skillful operators, and as a rule are eminently successful. One writes regretfully that their school is not open to women, but adds, "I wish that ten or twenty would make application at once."

The Ohio College of Dental Surgery reports that this college was the first to receive in its classes and graduate a woman dentist, and since that time it has had women among its students in nearly every session, and it is not an unusual thing for the highest honors to be accorded to the lady graduate.

One of the objections strongly urged against women dentists is, that their lack of muscular power disqualifies them for the work of extracting teeth. In reply I would state that in these days of progressive dentistry the extracting of teeth forms a small part of the work of an educated and conscientious dentist; instead of extracting, the greater number of the diseased and aching teeth are properly treated and filled. But in those cases where extracting becomes a necessity the members of the profession will agree with me in saying that the successful use of the forceps depends largely upon the skill of the operator, and not wholly, as is generally supposed, on one's physical strength. In proof of the fitness of women for this work I will cite the two convincing proofs.

First, there are now in the United States five dental colleges in which women receive equal opportunities for instruction as those offered to men, and further that this number is gradually increasing.

Second, that there are nearly one hundred women practicing in this country, while many have come here from Europe to fit themselves for this purpose, and have returned to their own country to practice their professional calling; and the practice of these dentists amount to sums varying from one to ten thousand dollars annually.

